# A Study on the Effects of Different Self‐Controlled Feedback Methods on the Learning of Table Tennis Serving Skills

**DOI:** 10.1002/ejsc.12324

**Published:** 2025-05-31

**Authors:** Wenlong Ma, Jingfeng Chen, Qing Yi, Shuai Zhang, Wei Xing, Honglie Ren, Rongzhi Li

**Affiliations:** ^1^ School of Physical Education Shanghai University of Sport Shanghai China; ^2^ Department of Health and Physical Education The Education University of Hong Kong Hong Kong China; ^3^ Faculty of Sports and Exercise Science University Malaya Kuala Lumpur Malaysia; ^4^ Institute of Physical Education and Training Harbin Sport University Harbin China; ^5^ College of Physical Education and Health Engineering Taiyuan University of Technology Taiyuan China; ^6^ Institute of Physical Education and Training Beijing Sport University Beijing China

**Keywords:** feedback methods, motor skill learning, self‐control, table tennis, task difficulty

## Abstract

This study explores how different self‐controlled feedback methods affect table tennis serving skills at various task difficulties. Using a self‐selection frequency technique, participants performed 10 rounds of serving practice with three feedback methods: KP, video, and video + KP. This study measured accuracy and skill evaluation of 120 first‐year students majoring in non‐physical education. In simple tasks, significant performance differences were observed between groups in immediate tests, but not in delayed tests. The video + KP method yielded the best outcomes, followed by video and KP. In complex tasks, performance differences were highly significant in both immediate and delayed tests, with video + KP showing superior results. Feedback request frequency trends were consistent across groups but varied with task complexity. For simple tasks, feedback had little impact on long‐term retention, whereas for complex tasks, differences were significant. Overall, self‐controlled feedback methods (KP, video, and video + KP) improved motor skill learning, with the video + KP method being most effective. The key contribution of this study lies in its innovative combination of self‐controlled feedback types, demonstrating that the integration of both video and KP feedback significantly enhances skill acquisition, particularly for complex tasks, offering practical implications for optimizing feedback strategies in sports education.

**Summary**

- Self‐controlled feedback significantly improves table tennis serve performance, especially under complex task conditions.
- Combining video and KP feedback produces the best learning outcomes compared to using either feedback method alone.
- The frequency of feedback requests is influenced by task complexity, with more frequent requests occurring in complex tasks.
- This study offers practical insights into optimizing feedback strategies in sports training.

## 1 Introduction

As a sport of net‐separated competition, table tennis features a wide variety of tactical and technical options and variations (Nikolakakis et al. 2021). Among them, serving techniques are considered one of the most critical performance indicators in table tennis. The serve is the starting point of a table tennis match (Katsikadelis et al. 2010), and it is also the only unforced technique in the game (Yu et al. 2007). Players with strong serving skills can gain the initiative or score directly during a match (Đukić and Ivanek 2020). An excellent table tennis player must possess outstanding strengths and no significant weaknesses. Without effective serving techniques, even if a player has powerful shots and strong spin capabilities, these features cannot be fully utilized (Talović et al. 2011). Therefore, effectively learning and improving table tennis serving skills is a critical aspect of athlete training. Traditional methods for enhancing table tennis skills primarily rely on repetitive physical and technical training (Sagala et al. 2024), which focuses on coaches' singular feedback and external observation during practice while neglecting the subjective experiences and feedback of the learners themselves. Feedback is a process in which the factors that generate results are modified, corrected, or reinforced (Mory 2013). It is a core research topic in the field of motor skill learning and has a significant impact on the effectiveness of skill acquisition (Wulf et al. 1998). In the domain of motor skill learning, feedback is typically categorized into intrinsic feedback and extrinsic feedback (Fiveable 2024). In recent years, self‐controlled feedback has emerged as a novel learning tool. Self‐controlled feedback refers to a mechanism that allows learners to autonomously choose the mode and timing of feedback during the learning process (Carter et al. 2014). Compared to traditional feedback methods, self‐controlled feedback enhances learners' initiative, providing them with a greater sense of control and responsibility during the learning process, thereby promoting the development of intrinsic motivation (Chiviacowsky et al. 2012). Self‐controlled feedback can be broadly categorized into two types: knowledge of performance (KP) feedback and knowledge of results (KR) feedback. KP feedback focuses on the performance aspects of motor skill execution. It allows learners to choose when to review their performance based on their individual needs. KP provides information on the movement process, for example, trajectory and pattern of force in time, and, by doing so, may complement or even substitute KR in certain tasks (Oppici et al. 2024). KR feedback primarily focuses on whether the learner's performance meets the expected outcomes (Aoyagi et al. 2019). Existing research has demonstrated that knowledge of results (KR) is a crucial factor in enhancing motor skill learning, such as how immediate feedback on outcomes can improve the performance of arm throwing speed in handball (Štirn et al. 2017). The third type of self‐controlled feedback combines KP and KR feedback, addressing both the learner's movement execution process and the feedback on outcomes. For example, after reviewing a video of their own serve, learners can choose to receive feedback from a coach or conduct self‐analysis, helping them identify issues in their technique and make adjustments. This approach aligns with augmented feedback in the field of motor skill learning, which encompasses both KP and KR feedback (Petancevski et al. 2022). Self‐selected frequency techniques, a variation of frequency‐modulation strategies within augmented feedback, allow learners to control the frequency of feedback, thereby tailoring the learning experience to their needs (Zhou and Yang 2002).

Video feedback provides objective performance data, such as key performance indicators, to assist coaches and athletes in making more precise decisions, thereby gaining a deeper understanding and mastery of the key factors influencing athletic performance (Wright et al. 2013). In the field of sports education, video feedback is used to support beginners in learning gymnastics (Potdevin et al. 2018). In table tennis research, video feedback is often applied in classroom teaching (Liang‐Bing 2020). The study of different combinations of feedback methods and their effects represents a new area of exploration in motor skill learning, with the combination of video feedback and KP feedback being a primary focus. Research has shown that receiving KP (error correction) feedback before watching video feedback can improve movement execution (Kernodle and Carlton 1992). Based on the theoretical approach emphasizing process control, self‐control and self‐regulation are considered equivalent concepts (Li et al. 2011). Self‐regulation refers to an individual's control over their psychological state, cognitive processes, and specific behaviors. It is one of the core functions of self‐awareness and an essential psychological process. It primarily involves the regulation of thoughts, emotions, impulses, and desires, as well as task performance and attentional processes (Huajian 2024). In the field of motor skill learning, self‐control is reflected in its integration with feedback frequency. Studies on the impact of self‐controlled knowledge of results (SCKR) and other schedules of knowledge of results (KR) at varying relative frequencies (25%, 50%, and 100% acquisition trials) on motor skill learning have shown that both self‐control and moderate‐frequency feedback enhance learning (Hebert and Coker 2021).

A review of previous research reveals that studies in this field have predominantly focused on fixed relative feedback frequencies, with limited exploration of self‐selected frequency techniques and inconclusive empirical results. Research on KR (knowledge of results) is more abundant, whereas studies on KP (knowledge of performance) are relatively scarce and primarily emphasize normative aspects, with insufficient discussion on operational accuracy. Studies on the impact of video feedback on motor skill performance mainly measure movement outcomes, with fewer investigations assessing relative movement structures and skill patterns. Only one study utilized the Game Performance Assessment Instrument (GPAI) to evaluate futsal skills, focusing on three aspects: decision‐making (correct or incorrect), skill execution (effective or ineffective), and support provision (correct or incorrect) (Hadiana et al. 2020). These dimensions address not only movement outcomes but also movement structures and skill patterns. Further research is needed on the combination of video and KP feedback methods. The effects on movement form (normativity) have not been adequately explained, and learning performance under varying task difficulty conditions remains insufficiently validated.

This study uses the serve (drive shot and backspin) in table tennis as the test skill and employs the self‐controlled frequency feedback technique to explore the impact of different self‐controlled feedback methods on the learning performance of serving skills at different task difficulties. The experimental hypotheses are as follows: (1) Regarding the hypothesis of self‐controlled feedback frequency: The trend of feedback frequency requests in different self‐controlled experimental groups is consistent and unrelated to the complexity of the task. The number of feedback requests (mean) in the same self‐controlled feedback method is related to the complexity of the task. (2) Regarding the hypothesis of different feedback methods on sports skill learning performance: KP is an effective way to improve sports skill learning and mastery, video is more effective than KP in improving sports skill learning and mastery, and the combination of video + KP is more effective than either video or KP alone in improving sports skill learning and mastery, regardless of task complexity. (3) The effect of using self‐controlled feedback methods on sports skill learning performance is related to the complexity of the task.

## 2 Methods

### 2.1 Participants

A total of 120 first‐year undergraduate students majoring in nonsports disciplines were recruited for this study. All participants were right‐handed players and were randomly assigned to 12 groups, with 10 participants in each group. The groups included the following: (1) simple task self‐controlled experimental groups (KP feedback, video feedback, video + KP feedback) and their corresponding control groups (3 groups), and (2) complex task self‐controlled experimental groups (KP feedback, video feedback, video + KP feedback) and their corresponding control groups (3 groups). All participants had no prior experience in similar experiments and were prohibited from engaging in any serving‐related practice outside of the experimental requirements during the study period.

### 2.2 Experimental Venue and Equipment

The experiment was conducted in a table tennis hall equipped with 12 table tennis tables, rackets, multiple table tennis balls, six digital video cameras, measuring rulers, computers, and designated target areas. The target areas were marked in the serving landing region to indicate different scoring zones based on the ball's placement.

### 2.3 Experimental Tasks

The simple task group practiced the forehand drive serve (simple task), whereas the complex task group practiced the forehand backspin serve (complex task). During the practice phase, participants in each group performed 10 rounds of serving practice according to the task requirements under different feedback conditions (no feedback, KP feedback, video feedback, and video + KP feedback). Each round lasted 2 min, with a 1‐min break between rounds. After the practice phase, all participants underwent a retention test involving 10 no‐feedback serves, first after a 10‐min rest and then after a 24‐h rest. Their performance in terms of RMSE (accuracy score and technical evaluation score) was recorded.

### 2.4 Experimental Design

The independent variables were KP feedback, video feedback, and video + KP feedback. The dependent variable was the operational performance RMSE of the forehand drive serve (simple task) and the forehand backspin serve (complex task). The self‐controlled experimental group could actively request feedback at any time based on their individual learning needs, whereas the control group underwent the experiment without any feedback. Participants were instructed to aim the ball as accurately as possible at the target area while maintaining proper serving form.

### 2.5 Experimental Procedure

**FIGURE 1:**
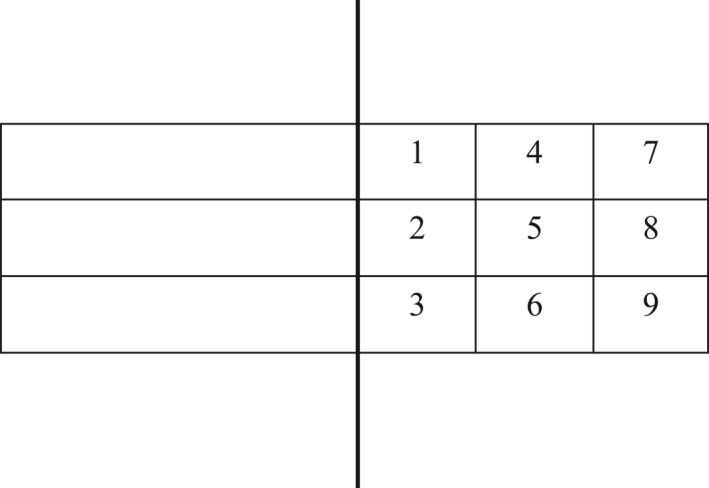
Target area division for right‐handed players.

- *Step 1*: All participants underwent a baseline skill test. The serving techniques were explained and demonstrated to familiarize participants with the experimental procedure. Each simple task group performed a baseline test for the forehand drive serve under no‐feedback conditions, whereas each complex task group did the same for the forehand backspin serve. RMSE (accuracy score and technical evaluation score) was recorded for each group.
- *Step 2*: During the practice phase, participants in the simple and complex task groups practiced the corresponding serving skills. After demonstrations, each group performed 10 rounds of practice (2 min per round with 1‐min breaks). Instructors provided KP feedback, video feedback, or video + KP feedback upon request for the self‐controlled groups and ensured control groups received the corresponding feedback passively. The organizers prepared standard videos for the forehand drive and backspin serves, recorded videos for the self‐controlled (video, video + KP) experimental groups, and tracked the feedback frequencies requested by self‐controlled groups. Experts recorded RMSE (accuracy and technical evaluation scores) for all groups during rounds 2, 4, 6, 8, and 10. Accuracy scores: The target area (half of the table) was divided into nine equally sized zones: 1 (forehand short), 2 (middle short), 3 (backhand short), 4 (forehand half‐table), 5 (middle half‐table), 6 (backhand half‐table), 7 (forehand long), 8 (middle long), and 9 (backhand long). Edge positions were assigned the highest difficulty scores (8 points for edge and adjacent areas), decreasing progressively to 1 for zones farther away. Net or out‐of‐bounds shots scored 0 points. Digital video cameras captured the target area and participants' movements throughout the experiment for precise evaluation. Technical evaluation scores: Table tennis specialists assessed the serving form on a scale of 0–10, with higher scores indicating better form. Each task group was assigned three experts, and the average score was used as the final score. As shown in Figure 1, the target area was divided into nine zones to evaluate the accuracy of the serve.
- *Step 3*: In the retention phase, all participants performed a retention test involving 10 no‐feedback serves for the forehand drive and backspin serves after a 10‐min rest and a 24‐h rest. RMSE (accuracy score and technical evaluation score) was recorded for all groups.

### 2.6 Statistical Analysis

The data utilized in this study were derived from an experimental project conducted at the China Table Tennis College, Shanghai University of Sport, between December 2024 and January 2025. The participants comprised first‐year undergraduate students enrolled in non‐physical education programs at the college. Prior to participation, all individuals underwent a standardized physical health screening and were confirmed to have no prior exposure to similar experimental protocols or specialized table tennis training, thereby ensuring a uniform baseline in motor skill proficiency across the sample. A cluster random sampling strategy was employed to achieve equivalency between the experimental and control groups with respect to gender, age, physical fitness, and initial table tennis skill levels. To enhance the reliability and external validity of the findings, strict control was maintained over experimental variables and operational procedures throughout this study. Motor skill performance was evaluated by a panel of experts with extensive experience in physical education and skill assessment, all of whom received standardized training to ensure interrater consistency. The entire experimental process was documented using high‐definition video recording equipment, and all critical data were subjected to double‐entry verification.

IBM SPSS Statistics version 29 (IBM Corp., Armonk, NY, US) was used to analyze the data. One‐way ANOVA was conducted to examine the differences in accuracy and technical evaluation scores under no‐feedback conditions during the practice phase for tasks of varying complexity. The one‐way ANOVA allowed researchers to assess the statistical differences in participants' performance across different task complexities and evaluate the impact of feedback methods on learning outcomes. Independent sample *t*‐tests were used to analyze accuracy and technical evaluation scores in the immediate (10‐min) and delayed (24‐h) retention tests. The independent sample *t*‐test was used to determine whether there were significant mean differences between the groups. This helped validate the effect of self‐controlled feedback methods on skill retention.

## 3 Experimental Results

### 3.1 Participants' Baseline Level

Under the no‐feedback condition, each group performed 10 trials of a simple task (flat forehand strokes) and 10 trials of a complex task (backspin serves). The baseline performance of each group (12 groups in total) showed that the differences in accuracy scores between the simple task and the complex task were not significant (as shown in Table 1). For the simple task, *F* = 0.961, *p* = 0.486; for the complex task, *F* = 0.262, *p* = 0.991. The differences in technique evaluation scores were also not significant (as shown in Table 2). For the simple task, *F* = 0.311, *p* = 0.990; for the complex task, *F* = 0.183, *p* = 0.839. These results indicate that there were no significant differences in the baseline operational skill levels between groups. Specifically, both the accuracy of movements and the technique evaluation scores did not show statistically significant differences (*p* > 0.05). Therefore, it can be concluded that all participants had similar initial skill levels in table tennis, meaning the baseline levels of the experimental groups were essentially the same.

**TABLE 1:** Differences in serving action accuracy scores under no‐feedback condition for different task difficulties during the practice phase.

Task difficulty	Group	*N*	Mean	Standard deviation	Significance	*F*
Simple task	Group 1	10	6.1	0.74	0.486	0.961
	Group 2	10	6.5	0.53		
	Group 3	10	6.5	0.85		
	Group 4	10	5.8	0.42		
	Group 5	10	6.4	0.52		
	Group 6	10	6.4	0.84		
	Group 7	10	6.1	0.57		
	Group 8	10	6	0.67		
	Group 9	10	6.1	0.88		
	Group 10	10	6.1	0.74		
	Group 11	10	6.1	0.74		
	Group 12	10	6.1	0.74		
	Total	120	6.2	0.70		
Difficult task	Group 1	10	5.6	0.97	0.262	0.991
	Group 2	10	5.9	0.88		
	Group 3	10	5.7	0.95		
	Group 4	10	5.5	0.85		
	Group 5	10	5.8	1.03		
	Group 6	10	6	0.67		
	Group 7	10	5.7	0.82		
	Group 8	10	5.7	1.34		
	Group 9	10	5.8	0.79		
	Group 10	10	5.9	0.57		
	Group 11	10	5.6	0.97		
	Group 12	10	5.8	0.63		
	Total	120	5.8	0.86		

**TABLE 2:** Differences in serving action evaluation scores under no‐feedback condition for different task difficulties during the practice phase.

Task difficulty	Group	*N*	Mean	Standard deviation	Significance	*F*
Simple task	Group 1	10	25	2.21	0.311	0.990
	Group 2	10	24.5	2.83		
	Group 3	10	25	0.67		
	Group 4	10	24.9	0.74		
	Group 5	10	24.8	0.63		
	Group 6	10	24.7	0.67		
	Group 7	10	24.9	0.74		
	Group 8	10	24.8	0.79		
	Group 9	10	25.2	2.10		
	Group 10	10	25.5	1.58		
	Group 11	10	24.9	0.74		
	Group 12	10	24.9	0.74		
	Total	120	24.9	1.37		
Difficult task	Group 1	10	21.6	2.63	0.183	0.839
	Group 2	10	21.5	2.68		
	Group 3	10	21.6	2.37		
	Group 4	10	21.7	0.67		
	Group 5	10	21.6	0.70		
	Group 6	10	21.5	0.71		
	Group 7	10	22	0.47		
	Group 8	10	21.9	0.57		
	Group 9	10	21.9	2.85		
	Group 10	10	22.2	2.53		
	Group 11	10	21.5	0.97		
	Group 12	10	22	0.67		
	Total	120	21.8	1.70		

### 3.2 Test Results During the Practice Phase

During the practice phase, the experiment organizers recorded the number of feedback requests made by participants in each self‐controlled experimental group for both simple and complex tasks during the second, fourth, sixth, eighth, and tenth trials. Simultaneously, expert evaluators conducted on‐site scoring of serving performance. Statistical analysis of the data yielded the frequencies of different self‐controlled feedback methods for different task difficulties (as shown in Table 3), technique evaluation scores (as shown in Table 4), and accuracy scores (as shown in Table 5). From Table 3, it can be observed that in terms of feedback frequency: For the simple task, the self‐controlled (KP) experimental group requested an average of 4.4 feedback instances, with a relative frequency of 31.0%. The self‐controlled (video) experimental group requested an average of 5 feedback instances, with a relative frequency of 35.2%. The self‐controlled (video + KP) experimental group requested an average of 4.8 feedback instances, with a relative frequency of 33.8%. For the complex task, the self‐controlled (KP) experimental group requested an average of 5.12 feedback instances, with a relative frequency of 28.2%. The self‐controlled (video) experimental group requested an average of 6.7 feedback instances, with a relative frequency of 37.0%. The self‐controlled (video + KP) experimental group requested an average of 6.3 feedback instances, with a relative frequency of 34.8%. The results indicate that the trend in the number of feedback requests was consistent across all self‐controlled experimental groups, regardless of task complexity. Specifically, the number of feedback requests was higher in the early stages of practice and gradually decreased as practice progressed. However, even in the later stages of practice, feedback requests did not drop to zero. The self‐controlled (video) experimental group had the highest average feedback frequency, followed by the self‐controlled (video + KP) experimental group, and then the self‐controlled (KP) experimental group, regardless of task complexity. The average feedback frequency of all self‐controlled experimental groups for complex tasks was generally higher than that of the self‐controlled experimental groups for simple tasks.

**TABLE 3:** The number of feedback instances for different self‐controlled feedback methods in different task difficulty levels during the practice phase.

Task category	Average number of feedback requests	Number of participants per group	Total feedback requests	Relative frequency (%)
Simple task self‐controlled (KP)	4.4	40	176	31.0
Simple task self‐controlled (video)	5	40	200	35.2
Simple task self‐controlled (video + KP)	4.8	40	192	33.8
Total	14.2	120	568	100.0
Complex task self‐controlled (KP)	5.1	40	204	28.2
Complex task self‐controlled (video)	6.7	40	268	37.0
Complex task self‐controlled (video + KP)	6.3	40	252	34.8
Total	18.1	120	724	100.0

**TABLE 4:** Mean technique evaluation scores for different self‐controlled feedback methods in tasks of varying difficulty during the practice phase.

Group	Feedback methods	Task complexity	Second round	Fourth round	Sixth round	Eighth round	Tenth round
Group 1	KP	Simple	12.5	23.0	23.1	23.5	23.3
Group 2	No feedback	Simple	6.4	17.6	18.3	22.6	22.7
Group 3	Video	Simple	13.5	23.6	23.7	24.0	24.2
Group 4	No feedback	Simple	11.4	16.3	18.5	23.4	24.0
Group 5	Video + KP	Simple	15.0	24.2	24.3	24.4	25.3
Group 6	No feedback	Simple	12.1	17.6	18.8	24.0	24.4
Group 7	KP	Complex	12.3	22.6	22.9	23.1	23.2
Group 8	No feedback	Complex	6.0	16.9	19.8	21.7	22.3
Group 9	Video	Complex	13.0	23.1	23.3	23.6	23.9
Group 10	No feedback	Complex	11.0	17.4	20.4	22.5	23.6
Group 11	Video + KP	Complex	14.3	23.6	24.0	24.3	25.0
Group 12	No feedback	Complex	11.7	17.2	18.5	23.0	24.1

*Note:* The odd‐numbered groups are assigned to the experimental group, whereas the even‐numbered groups are assigned to the control group.

**TABLE 5:** Mean technical evaluation scores of the 24‐h delayed retention test under different self‐controlled feedback modes for tasks of varying difficulty.

Group	Feedback methods	Task complexity	First round	*t*	*F*	*p*
Group 1	KP	Simple	22.8	1.071	0.007	0.291
Group 2	No feedback	Simple	21.9			
Group 3	Video	Simple	23.2	0.154	3.255	0.879
Group 4	No feedback	Simple	23.1			
Group 5	Video + KP	Simple	23.9	0.372	3.267	0.712
Group 6	No feedback	Simple	23.6			
Group 7	KP	Complex	21.3	2.930	0.138	0.006
Group 8	No feedback	Complex	19.5			
Group 9	Video	Complex	23.4	3.310	4.793	0.002
Group 10	No feedback	Complex	21.3			
Group 11	Video + KP	Complex	26.1	4.859	4.418	< 0.001
Group 12	No feedback	Complex	22.4			

*Note:* The odd‐numbered groups are assigned to the experimental group, whereas the even‐numbered groups are assigned to the control group.

From Table 4, it can be seen that in terms of technique evaluation scores, the trend in the changes of the mean technique evaluation scores for each self‐controlled experimental group is consistent and unrelated to task complexity. Specifically, the mean serving technique evaluation scores were lower in the early stages of practice and gradually increased as the experiment progressed, with the most significant improvement in the technique evaluation scores occurring in the 4th round. The self‐controlled (video + KP) experimental group had higher mean serving technique evaluation scores than the self‐controlled (video) experimental group, which in turn had higher scores than the self‐controlled (KP) experimental group, regardless of task complexity. For complex tasks, the mean serving technique evaluation scores for all self‐controlled experimental groups were generally lower than those for the simple task self‐controlled experimental groups. The self‐controlled (video + KP) experimental group for the complex task showed a significant improvement in mean serving technique evaluation scores in the 6th round, which was similar to the scores in the 4th round.

**FIGURE 2:**
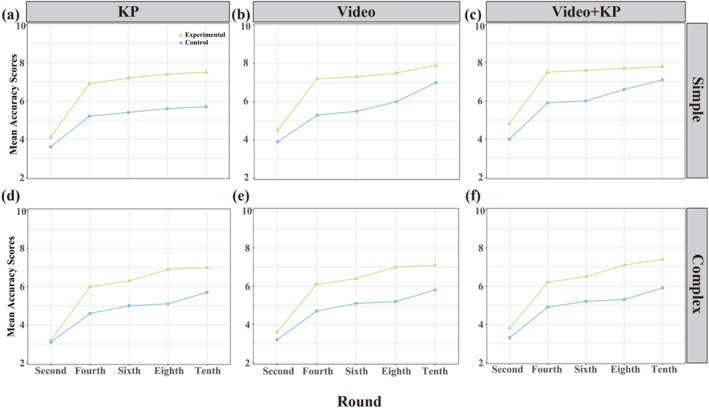
Mean accuracy scores for different self‐controlled feedback methods in tasks of varying difficulty during the practice phase. (a) KP mean accuracy scores in the simple task. (b) Video mean accuracy scores in the simple task. (c) Video + KP mean accuracy scores in the simple task. (d) KP mean accuracy scores in the complex task. (e) Video mean accuracy scores in the complex task. (f) Video + KP mean accuracy scores in the complex task.

From Figure 2, it can be seen that in terms of accuracy scores, the trend in the changes of mean accuracy scores for each self‐controlled experimental group is consistent and unrelated to task complexity. Specifically, the mean serving accuracy scores were lower in the early stages of practice and gradually increased as the experiment progressed, with the most significant improvement observed in the 4th round. The self‐controlled (video + KP) experimental group achieved higher mean serving accuracy scores than the self‐controlled (video) experimental group, which in turn scored higher than the self‐controlled (KP) experimental group, regardless of task complexity. For complex tasks, the mean serving accuracy scores of all self‐controlled experimental groups were generally lower than those of the simple task self‐controlled experimental groups. Significant improvements in mean serving accuracy scores were observed for the complex task self‐controlled (video + KP) experimental group and the simple task self‐controlled (video) experimental group in the sixth round, which were similar to the improvements in the fourth round.

### 3.3 Immediate Retention Test Results After 10 Min

**FIGURE 3:**
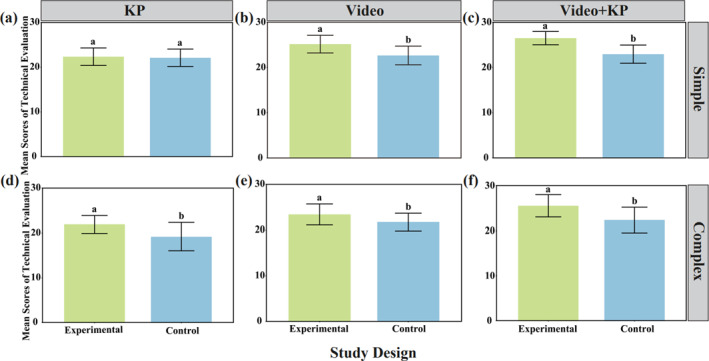
Mean scores of technical evaluation in the immediate retention test after 10 min across different task difficulties and self‐controlled feedback methods. (a) KP mean scores of technical evaluation in the simple task. (b) Video mean scores of technical evaluation in the simple task. (c) Video + KP mean scores of technical evaluation in the simple task. (d) KP mean scores of technical evaluation in the complex task. (e) Video mean scores of technical evaluation in the complex task. (f) Video + KP mean scores of technical evaluation in the complex task.

After a 10‐min rest, the average scores of the technical evaluation (mean values) for participants in simple and complex tasks in the immediate retention test are shown in Figure 3. For simple tasks, the difference between the KP experimental group and the control group was not significant (*t* = 0.406, *F* = 0.290, *p* = 0.687). The difference between the video experimental group and the control group was significant (*t* = 3.939, *F* = 0.681, *p* < 0.001). Similarly, the difference between the video + KP experimental group and the control group was significant (*t* = 5.815, *F* = 14.238, *p* < 0.001). The results of the immediate retention test scores (mean values) after 10 min indicate that, for learning simple tasks, the self‐controlled (KP) feedback method had minimal impact on the standardization of motor skills compared to the control group. However, the average technical evaluation scores of the self‐controlled (KP) experimental group were higher than those of the control group. In contrast, the self‐controlled (video) feedback method and the self‐controlled (video + KP) feedback method showed significant differences in their impact on the standardization of motor skills compared to the control group. Additionally, the average technical evaluation scores of both experimental groups were higher than those of the control group. For complex tasks, the difference between the KP experimental group and the control group was highly significant (*t* = 3.418, *F* = 4.712, *p* = 0.002). The difference between the video experimental group and the control group was also highly significant (*t* = 2.463, *F* = 0.958, *p* = 0.018). Likewise, the difference between the video + KP experimental group and the control group was highly significant (*t* = 3.740, *F* = 0.732, *p* < 0.001). The results of the immediate retention test scores (mean values) after 10 min indicate that, for learning complex tasks, the presence or absence of feedback had a substantial impact on the standardization of motor skills. Moreover, the average technical evaluation scores of all experimental groups were higher than those of the control group.

**FIGURE 4:**
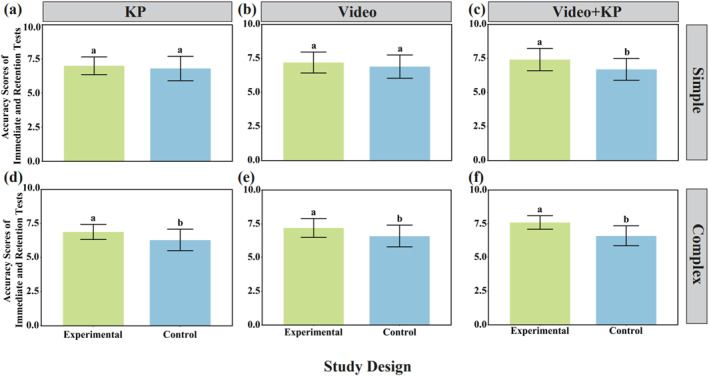
Accuracy scores of immediate and retention tests within 10 min under different self‐controlled feedback modes for tasks of varying difficulty. (a) KP accuracy scores of immediate and retention tests in the simple task. (b) Video accuracy scores of immediate and retention tests in the simple task. (c) Video + KP accuracy scores of immediate and retention tests in the simple task. (d) KP accuracy scores of immediate and retention tests in the complex task. (e) Video accuracy scores of immediate and retention tests in the complex task. (f) Video + KP accuracy scores of immediate and retention tests in the complex task.

After a 10‐min break, the accuracy scores (mean) of immediate serve performance in simple and complex tasks were tested, as shown in Figure 4. For the simple task, there was no significant difference between the KP experimental group and the control group, *F* = 8.444, *t* = 0.809, *p* = 0.424; no significant difference between the video experimental group and the control group, *F* = 0.378, *t* = 1.170, *p* = 0.249; but there was a significant difference between the video + KP experimental group and the control group, *F* = 0.031, *t* = 2.729, *p* = 0.010. The results of the 10‐min immediate retention test for accuracy scores (mean) indicated that in the simple task, compared with the control group, the effects of self‐controlled (KP) feedback and self‐controlled (video) feedback on motor skill stability were similar, but both experimental groups scored higher than the control group; however, the effect of self‐controlled (video + KP) feedback on motor skill stability was much greater compared to the control group, and the accuracy score (mean) of the video + KP experimental group was higher than that of the control group. For the complex task, there was a very significant difference between the KP experimental group and the control group, *F* = 5.108, *t* = 2.757, *p* = 0.009; a very significant difference between the video experimental group and the control group, *F* = 1.815, *t* = 2.494, *p* = 0.017; and a very significant difference between the video + KP experimental group and the control group, *F* = 5.400, *t* = 4.935, *p* < 0.001. The results of the 10‐min immediate retention test for accuracy scores (mean) indicated that in the complex task, the effect of feedback (or lack thereof) on motor skill stability was much greater, and the accuracy scores (mean) of all experimental groups were higher than those of the control group.

### 3.4 Results of the 24‐h Delayed Retention Test

After a 24‐h rest, the mean technical evaluation scores of participants for delayed serves in simple and complex tasks are shown in Table 5. For simple tasks, the differences between the KP experimental group and the control group were not significant, *F* = 0.007, *t* = 1.071, *p* = 0.291; the differences between the video experimental group and the control group were also not significant, *F* = 1.300, *t* = 1.301, *p* = 0.201 *F* = 1.300, *t* = 1.301, *p* = 0.201; similarly, the differences between the video + KP experimental group and the control group were not significant, *F* = 3.267, *t* = 0.372, *p* = 0.712. The mean results of the 24‐h delayed retention test suggest that in the learning of simple tasks, the presence or absence of feedback does not significantly impact the long‐term retention of motor skill accuracy. However, the mean technical evaluation scores of all experimental groups were higher than those of the control group. For complex tasks, the differences between the KP experimental group and the control group were highly significant, *F* = 0.138, *t* = 2.930, *p* = 0.006; the differences between the video experimental group and the control group were also highly significant, *F* = 4.793, *t* = 3.310, *p* = 0.002; and the differences between the video + KP experimental group and the control group were extremely significant, *F* = 4.418, *t* = 4.859, *p* < 0.001. The mean results of the 24‐h delayed retention test indicate that in the learning of complex tasks, the presence or absence of self‐controlled feedback has a highly significant impact on the long‐term retention of motor skill accuracy, and the mean technical evaluation scores of all experimental groups were higher than those of the control group.

After a 24‐h rest, the mean accuracy scores for delayed serves in simple and complex tasks for each group are shown in Table 6. For simple tasks, the differences between the KP experimental group and the control group were not significant, *F* = 0.084, *t* = 0.890, *p* = 0.379; the differences between the video experimental group and the control group were also not significant, *F* = 26.389, *t* = 1.219, *p* = 0.230; similarly, the differences between the video + KP experimental group and the control group were not significant, *F* = 1.179, *t* = 1.145, *p* = 0.259. The mean accuracy scores of the 24‐h delayed retention test indicate that in the learning of simple tasks, the presence or absence of feedback does not significantly affect the long‐term retention of motor skill accuracy. However, the mean accuracy scores of all experimental groups were higher than those of the control group. For complex tasks, the differences between the KP experimental group and the control group were highly significant, *F* = 5.692, *t* = 2.729, *p* = 0.010; the differences between the video experimental group and the control group were also highly significant, *F* = 0.010, *t* = 2.663, *p* = 0.011; and the differences between the video + KP experimental group and the control group were extremely significant, *F* = 0.028, *t* = 3.349, *p* = 0.002. The mean accuracy scores of the 24‐h delayed retention test suggest that in the learning of complex tasks, the presence or absence of self‐controlled feedback has a highly significant impact on the long‐term retention of motor skill accuracy, and the mean accuracy scores of all experimental groups were higher than those of the control group.

**TABLE 6:** Mean accuracy scores of the 24‐h delayed retention test under different self‐controlled feedback modes for tasks of varying difficulty.

Group	Feedback methods	Task complexity	First round	*t*	*F*	*p*
Group 1	KP	Simple	6.7	0.890	0.084	0.379
Group 2	No feedback	Simple	6.5			
Group 3	Video	Simple	7.2	1.219	26.389	0.230
Group 4	No feedback	Simple	6.9			
Group 5	Video + KP	Simple	7.9	1.145	1.179	0.259
Group 6	No feedback	Simple	7.4			
Group 7	KP	Complex	5.8	2.729	5.692	0.010
Group 8	No feedback	Complex	5.1			
Group 9	Video	Complex	6.3	2.663	0.010	0.011
Group 10	No feedback	Complex	5.8			
Group 11	Video + KP	Complex	7.1	3.349	0.028	0.002
Group 12	No feedback	Complex	6.4			

*Note:* The odd‐numbered groups are assigned to the experimental group, whereas the even‐numbered groups are assigned to the control group.

### 3.5 Results of the Simple Task Tests

In the context of simple tasks, the effects of different self‐controlled feedback modes on the technical evaluation scores of flat serve strokes were analyzed, as shown in Table 7. During the 10‐min immediate retention test, the difference between the self‐controlled (KP) experimental group and the self‐controlled (video) experimental group was significant, *F* = 0.002, *t* = −4.557, *p* < 0.001; and the difference between the self‐controlled (video) experimental group and the self‐controlled (video + KP) experimental group was also significant, *F* = 1.660, *t* = −2.121, *p* = 0.041. The mean technical evaluation scores of the 10‐min immediate retention test indicate that different self‐controlled feedback modes have a significantly different impact on the accuracy of motor skills. Furthermore, the self‐controlled (video + KP) feedback mode yielded better performance in flat serve stroke learning compared to the self‐controlled (video) feedback mode, which, in turn, outperformed the self‐controlled (KP) feedback mode. During the 24‐h delayed retention test, the difference between the self‐controlled (KP) experimental group and the self‐controlled (video) experimental group was not significant, *F* = 19.628, *t* = −1.627, *p* = 0.116; similarly, the difference between the self‐controlled (video) experimental group and the self‐controlled (video + KP) experimental group was not significant, *F* = 0.609, *t* = −1.170, *p* = 0.095. The mean technical evaluation scores of the 24‐h delayed retention test suggest that different self‐controlled feedback modes have little impact on the long‐term retention of motor skill accuracy. However, the self‐controlled (video + KP) feedback mode showed a trend of better performance in flat serve stroke learning compared to the self‐controlled (video) feedback mode, which again outperformed the self‐controlled (KP) feedback mode.

**TABLE 7:** Effects of different self‐controlled feedback modes on technical evaluation scores of flat serve strokes in experimental groups for simple task tests.

Test type	Feedback types	*N*	Mean	Standard deviation	*t*	*F*	*p*
10‐min immediate retention test	KP	20	22.35	1.95	−4.531	0.01	< 0.001
	Video	20	25.15	1.95			
10‐min immediate retention test	Video	20	25.15	1.95	−2.47	2.186	0.018
	Video + KP	20	26.5	1.47			
24‐h delayed retention test	KP	20	22.8	2.51	−0.563	0.362	0.577
	Video	20	23.2	2.55			
24‐h delayed retention test	Video	20	23.2	2.55	−0.888	1.147	0.380
	Video + KP	20	23.9	2.06			

In the context of simple tasks, the effects of different self‐controlled feedback modes on the accuracy scores of flat serve strokes were analyzed, as shown in Table 8. During the 10‐min immediate retention test, the difference between the self‐controlled (KP) experimental group and the self‐controlled (video) experimental group was not significant, *F* = 2.803, *t* = 0.890, *p* = 0.379; similarly, the difference between the self‐controlled (video) experimental group and the self‐controlled (video + KP) experimental group was not significant, *F* = 0.447, *t* = −0.796, *p* = 0.431. The mean accuracy scores of the 10‐min immediate retention test suggest that different self‐controlled feedback modes have little impact on the stability of motor skills. However, the self‐controlled (video + KP) feedback mode showed a trend of better performance in flat serve stroke learning compared to the self‐controlled (video) feedback mode, which, in turn, outperformed the self‐controlled (KP) feedback mode. During the 24‐h delayed retention test, the difference between the self‐controlled (KP) experimental group and the self‐controlled (video) experimental group was significant, *F* = 5.765, *t* = −2.484, *p* = 0.018; and the difference between the self‐controlled (video) experimental group and the self‐controlled (video + KP) experimental group was highly significant, *F* = 0.117, *t* = −4.114, *p* < 0.001. The mean accuracy scores of the 24‐h delayed retention test indicate that, compared to the self‐controlled (video + KP) feedback mode, the self‐controlled (video) feedback mode has a significantly smaller effect on the long‐term retention of motor skill stability. The self‐controlled (video + KP) feedback mode was significantly superior in enhancing flat serve stroke learning performance compared to the self‐controlled (video) feedback mode. Similarly, compared to the self‐controlled (KP) feedback mode, the self‐controlled (video) feedback mode had a more pronounced effect on motor skill retention, with better flat serve stroke learning performance observed in the self‐controlled (video) experimental group.

**TABLE 8:** Effects of different self‐controlled feedback modes on accuracy scores of flat serve strokes in simple task tests.

Test type	Feedback types	*N*	Mean	Standard deviation	*t*	*F*	*p*
10‐min immediate retention test	KP	20	7	0.65	−0.89	2.803	0.379
	Video	20	7.2	0.77			
10‐min immediate retention test	Video	20	7.2	0.77	−0.796	0.447	0.431
	Video + KP	20	7.4	0.82			
24‐h delayed retention test	KP	20	6.7	0.73	−2.484	4.717	0.018
	Video	20	7.2	0.52			
24‐h delayed retention test	Video	20	7.2	0.52	−4.114	0.117	< 0.001
	Video + KP	20	7.9	0.55			

### 3.6 Results of Complex Task Tests

In the context of complex tasks, the effects of different self‐controlled feedback modes on the technical evaluation scores of underspin serves were analyzed, as shown in Table 9. During the 10‐min immediate retention test, the difference between the self‐controlled (KP) experimental group and the self‐controlled (video) experimental group was highly significant, *F* = 0.493, *t* = −2.213, *p* = 0.033; and the difference between the self‐controlled (video) experimental group and the self‐controlled (video + KP) experimental group was also highly significant, *F* = 0.220, *t* = −2.774, *p* = 0.009. The mean technical evaluation scores of the 10‐min immediate retention test indicate that, compared to the self‐controlled (video + KP) feedback mode, the self‐controlled (video) feedback mode has a significantly smaller effect on the accuracy of motor skills. Furthermore, the self‐controlled (video + KP) feedback mode significantly outperformed the self‐controlled (video) feedback mode in improving underspin serve learning performance. In contrast, the difference between the self‐controlled (video) feedback mode and the self‐controlled (KP) feedback mode in terms of the impact on motor skill accuracy was not substantial, although the self‐controlled (video) feedback mode showed a trend of better underspin serve learning performance compared to the self‐controlled (KP) feedback mode. During the 24‐h delayed retention test, the difference between the self‐controlled (KP) experimental group and the self‐controlled (video) experimental group was highly significant, *F* = 0.545, *t* = −2.978, *p* = 0.005; and the difference between the self‐controlled (video) experimental group and the self‐controlled (video + KP) experimental group was extremely significant, *F* = 0.493, *t* = −3.983, *p* < 0.001. The mean technical evaluation scores of the 24‐h delayed retention test indicate that different self‐controlled feedback modes have a substantial impact on the long‐term retention of motor skill accuracy. Additionally, the self‐controlled (video + KP) feedback mode significantly outperformed the self‐controlled (video) feedback mode, which in turn significantly outperformed the self‐controlled (KP) feedback mode in improving underspin serve learning performance.

**TABLE 9:** Effects of different self‐controlled feedback modes on technical evaluation scores of underspin serves in complex task tests.

Test type	Feedback types	*N*	Mean	Standard deviation	*t*	*F*	*p*
10‐min immediate retention test	KP	20	21.9	1.97	−2.213	0.493	0.033
	Video	20	23.4	2.30			
10‐min immediate retention test	Video	20	23.4	2.30	−2.774	0.220	0.009
	Video + KP	20	25.5	2.48			
24‐h delayed retention test	KP	20	21.3	2.15	−2.978	0.545	0.005
	Video	20	23.4	2.30			
24‐h delayed retention test	Video	20	23.4	2.30	−3.983	0.493	< 0.001
	Video + KP	20	26.1	1.98			

In the context of complex tasks, the effects of different self‐controlled feedback modes on the accuracy scores of underspin serves were analyzed, as shown in Table 10. During the 10‐min immediate retention test, the difference between the self‐controlled (KP) experimental group and the self‐controlled (video) experimental group was not significant, *F* = 2.477, *t* = −1.510, *p* = 0.139; however, the difference between the self‐controlled (video) experimental group and the self‐controlled (video + KP) experimental group was highly significant, *F* = 0.779, *t* = −2.084, *p* = 0.044. The mean accuracy scores of the 10‐min immediate retention test suggest that, compared to the self‐controlled (video + KP) feedback mode, the self‐controlled (video) feedback mode has a significantly smaller effect on the stability of motor skills. Furthermore, the self‐controlled (video + KP) feedback mode significantly outperformed the self‐controlled (video) feedback mode in improving underspin serve learning performance. In contrast, the difference between the self‐controlled (video) feedback mode and the self‐controlled (KP) feedback mode in terms of the impact on motor skill stability was not substantial, although the self‐controlled (video) feedback mode showed a trend of better underspin serve learning performance compared to the self‐controlled (KP) feedback mode. During the 24‐h delayed retention test, the difference between the self‐controlled (KP) experimental group and the self‐controlled (video) experimental group was significant, *F* = 0.010, *t* = −2.663, *p* = 0.011; and the difference between the self‐controlled (video) experimental group and the self‐controlled (video + KP) experimental group was highly significant, *F* = 0.177, *t* = −3.899, *p* < 0.001. The mean accuracy scores of the 24‐h delayed retention test indicate that different self‐controlled feedback modes have a significant impact on the long‐term retention of motor skills. Additionally, the self‐controlled (video + KP) feedback mode significantly outperformed the self‐controlled (video) feedback mode, which in turn significantly outperformed the self‐controlled (KP) feedback mode in improving underspin serve learning performance.

**TABLE 10:** Effects of different self‐controlled feedback modes on accuracy scores of underspin serves in complex task tests.

Test type	Feedback types	*N*	Mean	Standard deviation	*t*	*F*	*p*
10‐min immediate retention test	KP	20	6.9	0.55	−1.510	2.477	0.139
	Video	20	7.2	0.70			
10‐min immediate retention test	Video	20	7.2	0.70	−2.084	0.779	0.044
	Video + KP	20	7.6	0.50			
24‐h delayed retention test	KP	20	5.8	0.62	−2.663	0.010	0.011
	Video	20	6.3	0.57			
24‐h delayed retention test	Video	20	6.3	0.57	−3.899	0.177	< 0.001
	Video + KP	20	7.1	0.72			

## 4 Experimental Discussion

### 4.1 Overall Impact of Self‐Controlled Feedback on Learning Performance

Self‐controlled feedback significantly enhances learners' mastery of motor skills, demonstrating learning performance markedly superior to that of the control group (Post et al. 2016). By increasing learners' autonomy and engagement, self‐controlled feedback helps them focus more on and better understand the provided feedback, thereby facilitating effective skill acquisition (Wulf 2007). This can be understood from the perspective of self‐regulated learning (SRL), a concept in cognitive psychology that emphasizes learners' ability to take control of their learning process, including goal setting, self‐monitoring, and reflection on their performance. Self‐controlled feedback aligns with key principles of SRL. By allowing learners to choose the frequency and type of feedback they receive, this approach enhances their sense of self‐efficacy and agency, core elements of SRL. This study showed that using different feedback modes improved learners' skill mastery in both simple and complex tasks. Self‐controlled feedback affects learning outcomes in several ways. (1) Enhancing learners' sense of control: Self‐controlled feedback enables learners to experience greater control during the learning process, increasing their active participation and engagement. In this study, learners could choose the frequency and type of feedback based on their individual needs, thereby improving their autonomy and task focus. (2) Personalized feedback and goal orientation: Self‐controlled feedback allows learners to select feedback modes according to their personal learning goals and needs, achieving a more personalized learning experience. This individualized approach not only helps learners establish clearer goals for skill mastery but also enables them to adjust their learning strategies based on the feedback, making the skill acquisition process more efficient. For instance, KP feedback provides specific details about movements to help learners refine their actions, whereas video feedback offers visual playback, enabling learners to better understand their performance and identify issues. (3) Improving the reception and processing of feedback: Self‐controlled feedback is not just about allowing learners to choose the frequency and type of feedback during the learning process but also emphasizes the effective reception of feedback. When learners themselves decide when and how to receive feedback, their ability to process and accept the feedback improves. The findings of this study validate the critical role of self‐controlled feedback in enhancing learners' learning efficiency and skill mastery. Whether in the simpler flat serve task or the more complex underspin serve task, self‐controlled feedback consistently resulted in better learning outcomes compared to the control group. In summary, this study demonstrates that self‐controlled feedback in motor skill learning improves learners' skill mastery through multiple mechanisms and enhances their motivation and learning autonomy. The innovation of this study lies in the first‐time integration of three types of self‐controlled feedback—knowledge of performance (KP), video feedback, and the combination of video + KP feedback—into the learning of table tennis skills, further advancing the research on feedback strategies in motor skill acquisition.

### 4.2 The Impact of Task Difficulty on the Effectiveness of Self‐Controlled Feedback

Studies have indicated that providing feedback when performance is good is more effective for motor skill learning than giving feedback during poor performance (Chiviacowsky and Wulf 2007). This study further reveals the significant impact of task difficulty on the effectiveness of self‐controlled feedback, particularly in how different types of feedback perform under varying task difficulties. In the learning of simple tasks (e.g., flat serve) and complex tasks (e.g., underspin serve), the feedback effects differ, primarily because of the varying demands for feedback content and the effectiveness of feedback types depending on task complexity. Feedback effects in simple tasks: In simple tasks, although there was no significant difference in learning performance between the self‐controlled feedback (KP) group and the control group, the technical evaluation scores and accuracy scores of the self‐controlled feedback (KP) group consistently exceeded those of the control group. This suggests that although feedback forms are important in the learning of simple tasks, their overall impact is relatively minor. Simple tasks typically involve fewer skill elements and lower movement complexity, allowing learners to master most skills within a short time through basic technical training. Therefore, although feedback aids in task mastery, its effects are less pronounced in simple tasks. Nevertheless, even in simple tasks, self‐controlled feedback (KP) played an important role in fine‐tuning movements and improving skill precision. The key advantage of KP feedback lies in its ability to provide learners with specific movement instructions, helping them correct errors and refine technical details (Oppici et al. 2024). As a result, although the overall learning performance differences in simple tasks may not be significant, KP feedback still aids learners in mastering technical essentials with greater precision. Feedback is particularly critical in complex tasks, which involve more steps and higher skill requirements. Learners must integrate multiple sources of information to complete these tasks. In such tasks, the learning outcomes of the self‐controlled feedback (video + KP) group were significantly better than those of the other groups, highlighting the effectiveness of a diversified feedback combination for learning complex tasks. This result confirms the importance of feedback multidimensionality in learning complex tasks. Video feedback provides learners with a visual representation of their actions, helping them quickly identify errors and adjust strategies. Meanwhile, KP feedback offers verbal guidance, enabling learners to make detailed adjustments. Task complexity and feedback strategy alignment: Learning complex tasks requires learners to go beyond simple technical operations, demanding greater cognitive resources for skill integration and application. Against this backdrop, the combined effect of self‐controlled feedback (video + KP) is particularly evident. Video feedback enhances learners' understanding of the movement process through visual feedback, whereas KP feedback offers detailed guidance, allowing for precise adjustments and more effective task completion. This aligns with previous studies, demonstrating that the combination of visual feedback and verbal feedback significantly enhances learners’ mastery of motor skills in complex tasks (Akıncı 2004).

### 4.3 Research Limitations and Future Directions

This study, although achieving certain results in exploring the impact of different self‐controlled feedback methods on the learning performance of table tennis serving skills, has several limitations. First, the sample included only 120 first‐year college students who were nonsports majors, all of whom were right‐handed players. This limits the generalizability of the findings. Future research could consider expanding the sample size and including more diverse groups, such as athletes of different genders, skill levels, or sports majors, to enhance the broader applicability of the conclusions. Second, this study focused only on two tasks: forehand drive serve and forehand underspin serve. Although these tasks varied in difficulty, the limited range of task types may not fully reflect the effects of different feedback methods. Future research could include a wider variety of motor skill tasks to broaden the scope and depth of the investigation. Third, although this study examined the effects of KP feedback, video feedback, and their combination, it overlooked other types of feedback that might influence motor skill learning (e.g., KR feedback, real‐time feedback). Future studies could explore more diverse feedback formats and their combinations to comprehensively evaluate the effectiveness of self‐controlled feedback. Additionally, the time span of this study was relatively short, assessing skill retention through immediate testing (10 min post‐task) and delayed testing (24 h post‐task). Such a short time frame might not fully reflect the long‐term retention of skills. Subsequent research could adopt longer‐term follow‐up testing to examine the enduring effects of feedback methods on skill retention. Lastly, this study did not consider individual differences among feedback providers (e.g., teaching experience, communication skills), which could influence the effectiveness of feedback. Future research could further investigate the impact of provider factors on motor skill learning. Moreover, the experiment was conducted in a controlled environment, whereas real sports scenarios might involve more external interferences. Future studies could consider applying self‐controlled feedback in more complex and dynamic real‐world settings to validate its effectiveness. Meanwhile, the tracking period should be extended (e.g., by 1 week or 1 month) in order to more comprehensively assess the long‐term retention of skills. In conclusion, although this study provides valuable evidence regarding the effectiveness of self‐controlled feedback, further exploration is needed to evaluate its application in broader contexts.

## 5 Conclusion

This study investigated the effects of various self‐controlled feedback methods on the learning of table tennis serving skills. The findings reveal that although feedback has minimal impact on the long‐term retention of simple motor skills, it plays a critical role in enhancing the mastery of more complex skills. Specifically, the combination of video feedback and knowledge of performance (KP) feedback proved to be the most effective in improving both the accuracy and technique of learners, particularly in tasks of higher complexity. This suggests that a multidimensional feedback approach—integrating visual and corrective cues—can better facilitate motor skill acquisition in more complex tasks. In practical terms, coaches and instructors should consider incorporating both video and KP feedback into training regimens, especially when teaching intricate skills, to maximize learning outcomes. Future research should explore the impact of self‐controlled feedback across different sports and skill types, as well as examine the effects of longer‐term feedback strategies. Additionally, studies could focus on individual differences in feedback preferences to tailor learning experiences more effectively and improve overall performance retention.

## Ethics Statement

This study was carried out with the written informed consent of all participants and with the approval of the institutional review board of the exercise and health sciences department of Beijing Sport University, ensuring ethical standards were strictly adhered to throughout the research process.

## Conflicts of Interest

The authors declare no conflicts of interest.

## Data Availability

Data are available upon request from the corresponding author.
